# Modern Sociology

**Published:** 1899-10-28

**Authors:** 


### Modern Sociology.

### THE FALLACY OF STATISTICS.—THE "IMPASSE" AT BRUSSELS.

By a Correspondent.

Doctors are proverbially supposed to differ, but there are certain subjects upon which they are understood to be agreed. And ten years ago certainly the question of the medical regulation of the social evil was one of them. Hence, when the Congress of Social Hygiene assembled at Brussels a battle royal was anticipated between a solid phalanx of medical opinion in favour of regulation upon the one hand, and an enthusiastic body of "abolitionists" opposed to it upon moral and sentimental grounds on the other. And the question was put before the meeting squarely and frankly at the earliest possible moment, "Does the regulation of prostitution and medical examination of the prostitute diminish the prevalence of venereal disease?" Over this the din of battle raged for nearly two whole days, and then the President decided that it was inadvisable to put the question! The delegates found themselves in the position of Mr. Kipling upon a similar problem — the more they considered it the less they were able to come to any conclusion whatever. And the hottest part of the conflict was not, as had been dreaded by some, between medical experts on the one hand and enthusiastic but unscientific lay philanthropists on the other, but within the ranks of the profession itself. A majority of those who spoke were in favour of the traditional view as to the efficacy of regulation, but a vigorous and considerable minority entirely dissented from this.

The most surprising feature of the discussion was the appalling discrepancies between, and uncertainty of the statistics quoted by the two sides. There appeared to be no possibility of agreement in even the simplest statements of facts. Twice in the course of the debate was the experience of a single town quoted by speakers residing in it as supporting both views of the problem —regulation according to one, abolition according to the other. In Strasburg the statistics of a civilian medical officer varied widely from those of the military medical authorities. In fact, the confusion became so great that finally one speaker frankly blurted out that the "human element" in statistics had to be considered, and that unless he believed in a man he wasn't going to accept his figures. And dear old Fournier capped the climax by declaring that, for his part, if he was obliged to choose between statistics, he should take those that favoured the "reasonable view," viz., regulation.

There appeared to be no agreement upon even such simple questions of arithmetic, as whether syphilis was most prevalent per 1,000 population in London, Paris, or Berlin. Indeed, any attempt to institute such comparisons led to an exchange of international courtesies. A son of perfidious Albion declared that the disease was far more common in regulated Paris than in unregulated London. To which a prominent Parisian cuttingly retorted that if it was unusually prevalent in Paris it was due to the large number of foreigners who came there. Attracted by its delightful immorality and the alleged freedom from the consequences of vice, rejoined the Briton! And this between grave and reverend seigneurs of our honourable profession.

Trivial as the incident is, it furnishes the key to the situation. Almost the whole uncertainty and absence of reliable data which befog this subject depend upon the fact that the diseases in question are universally looked upon as something to be ashamed of, and are concealed or denied in every possible manner, so that statistics concerning them are not to be trusted. No scientist even, scarcely a medical man, will admit its occurrence in greater frequency than elsewhere in his own town or country—at least, not before foreigners. And, of course, the first and most vital question to be asked about regulation of any sort is, "Does it diminish the presence of disease?"

What have we actually to base our answer upon? Statistics which, bias apart, can at best claim only to represent from one-third to half the amount of disease which actually exists. In the first place nearly half the cases of gonorrhœa, and a respectable fraction of those of syphilis, never come under the care or even notice of a qualified physician at all. So fearful are they of detection that they resort to quack remedies, "sure cures," or to the recipes of boon companions of greater experience in afflictions of this sort. Then, of those who consult a physician, how many could we, under any circumstances, report or record? How many *dare* we, even if we would? The question answers itself. All that we have to depend on is the record books of hospitals, and these, also, are avoided like poison by a considerable proportion of this sort of case, even among the class which frequents their waiting-rooms. Even military statistics are vitiated to some degree by this same defect, unless physical inspection be carried out regularly every few weeks, a thing so bitterly resented as to be almost impracticable.

Thus we are faced with the fact that it is impossible to speak with any certainty whatever as to whether the amount of disease has diminished or increased 10, 20, or 30 per cent. under regulation upon a basis of statistics covering at best only 50 or 60 per cent. of the actual cases. So that each observer gathers such statistics as he can and estimates the remainder. And estimates, as Fournier frankly confessed, have a sad tendency to conform to the previous opinions of the "estimator."

We are in precisely the same quandary as with respect to the sister question, "Does regulation diminish the amount of prostitution?" It can easily be shown to diminish the number of those who are licensed and of recognised houses, but this proves little. To these women publicity and restraints carry nothing but disadvantage, and they evade the eye of the police as swiftly as possible, to sink into the dim and half-buried realm of clandestine but none the less real immorality, within whose borders are contained, even in the best regulated cities, from half to two-thirds of the unchaste women of the community.

As to their numbers we have absolutely nothing but conjecture and general estimates to guide us, and even experts cannot agree within tens of thousands in cities like London and Paris. So that we seem as far from mathematically exact statements of the extent of the evil and the prevalence of its consequences among us as we were fifty years ago. The very basis of the statistical method is correct observation of units, but in this matter the units are largely matters of guesswork.

